# N-terminal entrance loop of yeast Yps1 and *O*-glycosylation of substrates are determinant factors controlling the shedding activity of this GPI-anchored endopeptidase

**DOI:** 10.1186/s12866-015-0380-1

**Published:** 2015-02-26

**Authors:** Alexandre K Dubé, Marc Bélanger, Isabelle Gagnon-Arsenault, Yves Bourbonnais

**Affiliations:** Département de biochimie, microbiologie et bio-informatique, Institut de biologie intégrative et des systèmes and Regroupement PROTEO, Université Laval, Québec, QC Canada

**Keywords:** Fungal, Yapsin/N-entrance loop/Zymogen activation/GPI-protein, Shedding/post-ER, Quality control/*O*-glycosylation

## Abstract

**Background:**

*S. cerevisiae* Yps1 is the prototypical aspartic endopeptidase of the fungal yapsin family. This glycosylphosphatidylinositol (GPI) anchored enzyme was recently shown to be involved in the shedding of the GPI proteins Utr2, Gas1 and itself. It was also proposed to be part of a novel quality control mechanism that eliminates excess and/or misfolded GPI proteins. What regulates its shedding activity at the cell surface is however poorly understood. Yps1 is initially synthesized as a zymogen requiring proteolytic activation to remove a pro-peptide and further processing within a large insertion loop (N-entrance loop) generates a two-subunit endopeptidase. To investigate the role of this loop on its shedding activity, which typically takes place within Ser/Thr-rich domains, it was replaced with the short peptide found at the analogous position in Yps3. We also tested whether *O*-glycosylation might protect against proteolytic processing by Yps1.

**Results:**

We show here that replacement of the N-entrance loop (N-ent loop) of Yps1 generates a single chain endopeptidase that undergoes partial (pH 6.0) or complete (pH 3.0) pro-peptide removal. At both pH, the shedding activity of the chimeric endopeptidase (Yps1-DL) toward Gas1 and itself is strongly and drastically increased, respectively. A direct correlation between endoproteolytic cleavage of this loop in native Yps1 and its shedding is observed. The Yps1-dependent shedding of two model GPI proteins (Gas1 and Yps1) is also stimulated by the absence of the *O*-mannosyltransferases, Pmt4 and Pmt2 respectively, involved in *O*-glycosylation of their Ser/Thr-rich domains. Under these conditions, some Yps1-independent shedding is also observed.

**Conclusions:**

Partial pro-peptide removal is essential to produce a functional Yps1 endopeptidase. The Yps1 N-ent loop plays a major role in regulating the shedding activity of the endopeptidase, most likely by limiting access to the active site, and its cleavage in native Yps1 is associated with its shedding. *O*-glycosylation protects against Yps1-dependent and -independent shedding of GPI proteins. It is postulated that hypoglycosylation of cell surface proteins, which may occur for misfolded proteins that escaped the ER-associated degradation, might target their elimination through shedding by Yps1 and possibly other yapsin members.

## Background

Fungal yapsins constitute a special class of aspartic peptidases characterized by their attachment to membranes through a GPI anchor (see [1] for a review). The first and prototypical yapsin member Yps1 (E.C. 3.4.23.41) was discovered in *S. cerevisiae* because of its ability to process the α-factor precursor C-terminal to Lys-Arg cleavage sites when overexpressed in a *kex2Δ* mutant [2]. Soon after, it was demonstrated that Yps1 also recognizes and cleaves C-terminal to single basic residues (Lys or Arg) in heterogeneous model peptide substrates [3-6]. Typically, several members of the yapsin endopeptidases are expressed per fungal species and they all localize at the cell surface, either associated with the plasma membrane or the cell wall [1]. Although their functional significance has yet to be fully elucidated, *S. cerevisiae* Yps1 was shown to be involved in the ectodomain shedding of the transmembrane protein Msb2 [7] and of GPI-anchored proteins including the glucanosyltransferase Gas1, the chitin transglycosylase Utr2 and itself [8,9]. Furthermore, cleavage of GPI-Utr2 was proposed to constitute a novel cellular response to eliminate excess and/or unfolded GPI-proteins [9]. This hypothesis was recently further substantiated by a high-throughput experiment which revealed that a subset of yeast mutants known to trigger the unfolded protein response (UPR) are associated with an increased shedding of both Gas1 and Yps1^a^. What controls the activity of Yps1 and prevents undesirable shedding of important, correctly folded, GPI proteins? Because most aspartic peptidases are initially synthesized as inactive zymogen precursors [10], the activation mechanism of Yps1 possibly provides an important level of regulation. Another aspect that may control or limit the action of Yps1 at the cell surface is the nature of the substrate and, more specifically, the context in which the putative cleavage site lies. For instance, shedding of Gas1, Yps1 itself and Utr2 is known to occur within S/T-rich domains or clusters that are typically heavily *O*-glycosylated [8,9]. Could the extent of *O*-mannosylation in close vicinity of the cleavage site affect its recognition and/or hydrolysis by Yps1?

As revealed by previous studies using either a soluble truncated form of Yps1 [11,12] or native GPI-anchored Yps1 [8], the activation mechanism of this endopeptidase is complex and involves multiple proteolytic processing steps. The pioneer study of Cawley *et al.* [11] demonstrated that a unique feature of Yps1 is the cleavage of an internal loop located between Cys^117^ and Cys^186^ (Yps1 numbering) that is absent from the prototypical human pepsin A enzyme [E.C.3.4.23.1]. This cleavage generates a two-subunit endopeptidase (α and β), each subunit contributing an Asp residue of the catalytic site. For the native GPI-anchored enzyme, cleavage of this loop was shown to be strictly autocatalytic [8]. Furthermore, alternative processing sites were used to generate the two-subunit endopeptidase depending on the extracellular pH suggesting that this processing step occurs once the enzyme has reached the cell surface. The role of this loop and the significance of its cleavage on Yps1 function are at present not clear. An insertion loop at this position is also present in most fungal secreted aspartic peptidases (SAP) [13], including the yapsins [1], and structures from several SAPs suggest that it restricts access to the non-prime side of the binding pocket and, accordingly, was given the name ‘N-entrance loop’ (N-ent loop) [13-17]. Assuming that this loop also restricts access to the active site in the case of Yps1, and because cleavage of the loop is a late event, we hypothesized that it could be a prerequisite for its shedding activity [8]. However, experimental evidence supporting this hypothesis is lacking. In the present study, we engineered both a GPI-anchored and a soluble truncated version of Yps1 where the N-ent loop (residues 118 to 185 of Yps1) is replaced by the short peptide segment found at this location in the Yps1 homologue Yps3 (GSVMD). In addition to assessing directly the role of the N-ent loop in the shedding activity of Yps1, by generating a single subunit endopeptidase, this also facilitated the analysis of zymogen activation and its pH dependency.

*O*-mannosylation of proteins has been described as conferring stability to yeast cell surface proteins [18], and we therefore also addressed whether this modification could interfere with shedding by Yps1. In yeast, *O*-mannosylation on Ser and Thr residues is catalyzed by a family of *O*-mannosyltransferases, the Pmt enzymes [19]. Using Yps1 and Gas1 as model GPI substrates, we compared their shedding in the presence or absence of the Pmt primarily responsible for their *O*-mannosylation.

Our results clearly demonstrate that pro-peptide removal, even if only partial at pH 6.0, is essential to generate a functional Yps1 endopeptidase. We also show that replacement of the Yps1 N-ent loop to generate a single chain endopeptidase dramatically increases the release of Yps1 into the medium. This suggests that in native Yps1, this loop plays a determinant role in regulating its shedding activity. Finally, we observe increased amounts of Yps1 and Gas1 into the medium upon disruption of *PMT2 and PMT4,* respectively*,* the *O*-mannosyltransferases mainly involved in their *O-*glycosylation. It is postulated that hypoglycosylation may play a role in the elimination of misfolded proteins that escaped the ER quality control and reached the cell surface.

## Results

### N-ent loop mutant of GPI-Yps1 is active *in vivo*

**Figure 1:**
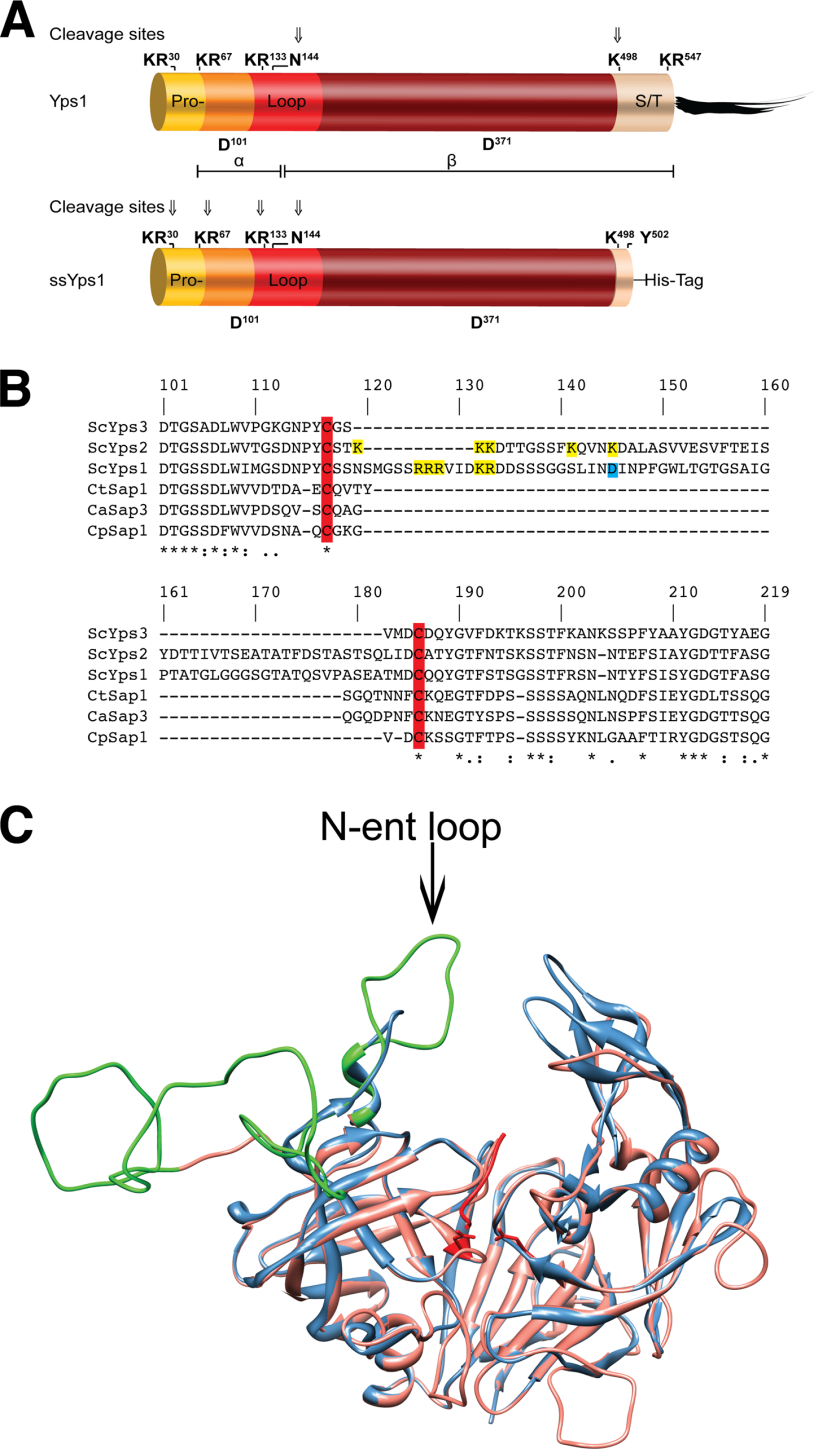
**Alignment and structure of ScYps1 vs CaSap3. A)** Schematic representation of native Yps1 and ssYps1 (adapted from Gagnon-Arsenault *et al.,* [8]). Cleavage sites known to be used in each forms are indicated by arrows. **B)** ClustalW alignment [20] of *S. cerevisiae* yapsins Yps3 (ScYps3; NP_013222.1), Yps2 (ScYps2; NP_010428.3) and Yps1 (ScYps1; NP_013221.1) with Secreted Aspartyl Proteinases Sapt1 from *C. tropicalis* (CtSap1; Q00663.1), Sap3 from *C. albicans* (CaSap3; XP_723210.1) and Sapp1 from *C. parapsilosis* (CpSap1; ACL81524.1) displaying the insertion loop located between the conserved cysteine residues (highlighted in red). The basic residues (highlighted in yellow) present in the insertion loop of ScYps1 and ScYps2 are shown along with the known N-terminus of the fastest migrating form of Yps1 β subunit (highlighted in blue). **C)** Modelled chimeric structure of ScYps1 (residues 76 to 490, in blue) and CaSap3 (residues 66 to 398, in orange) obtained from the ModWeb server. The N-ent loop (indicated by the arrow) of Yps1 is shown in green. The aspartic residues of the catalytic center and the inhibitor Pepstatin A are shown in red.

As cartooned in Figure 1A, full length Yps1 (top panel) and its truncated secreted version ssYps1 (bottom panel) possess a N-terminal propeptide that is known to be cleaved at the dibasic sites KR^30^ and KR^67^ in ssYps1 [8,11]. While removal of the pro-peptide is suspected to proceed similarly for full length Yps1, the exact N-terminus of the mature GPI-anchored endopeptidase has, however, not been determined. Both ssYps1 and Yps1 are also cleaved within an insertion loop to generate an α/β two-subunit endopeptidase [8,11]. Under acidic conditions, the fastest migrating isoform of the β subunit was shown to start at D^145^ suggesting either autocatalytic cleavage C-terminally to N^144^ or cleavage at basic sites upstream (*e.g.* KR^133^ which was determined for the slow migrating isoform of the ssYps1 β subunit [8]) followed by trimming by exoproteolysis as initially suggested by Olsen *et al.* [12]. Shedding of native Yps1 was shown to proceed through autocatalytic cleavage after K^498^ and, as the resulting short peptide (16 kDa) beginning with the sequence ^499^APGY was secreted, it strongly suggested further proteolytic processing at the only other basic site (KR^547^) prior to the attachment of the GPI anchor at N^548^ [8].

Alignment of Yps1 (ScYps1) with other *S. cerevisiae* yapsin members (ScYps2 and ScYps3) and secreted aspartic peptidases (SAP) from *C. tropicalis* (CtSap1), *C. albicans* (CaSap3) and *C. parapsilosis* (CpSap1), revealed that the insertion loop located between a conserved disulfide bridge is significantly larger in ScYps1 and ScYps2p (Figure 1B). As suggested by the chimeric structural model obtained by the ModWeb server [21,22] with ScYps1 and CaSap3 (Figure 1C), this loop is superimposed with the so-called N-ent loop of CaSap3, a second active site flap, which is believed to control access of the substrate to the active site cleft [15]. To investigate the functional role of this N-ent loop in Yps1 activity, a mutant (Yps1-DL) where this loop was replaced by the pentapeptide GSVMD found in ScYps3 was constructed.

**Figure 2:**
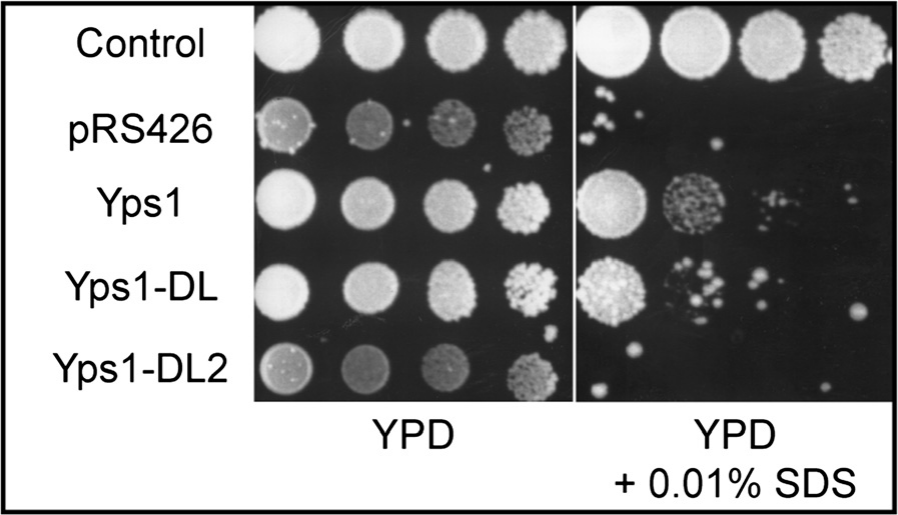
**Both native Yps1 and Yps1-DL correct the SDS hypersensitivity of** ***kex2Δ*** **yeast.** *yps1Δ* yeast transformed with pRS426 (Control) or *kex2Δ yps1Δ* yeast transformed with the indicated plasmids were first grown overnight in SC medium. Then, serial 1/5 dilutions were spotted onto YPD or YPD supplemented with 0.01% SDS and colonies were grown for 48 h at 30°C.

Overexpression of native Yps1 was previously shown to partially correct the SDS hypersensitivity of *kex2Δ* mutant yeast [8]. As shown in Figure 2, correction of this phenotype was comparable with the native form (Yps1) and the loop mutant (Yps1-DL) of Yps1, when overexpressed. In contrast, an active site mutant (D^101^E) of Yps1-DL (Yps1-DL2) was as inefficient as the plasmid alone (pRS426) in alleviating the *kex2*Δ SDS hypersensitivity. These results therefore indicated that deletion of the N-ent loop is not deleterious for the activity of the endopeptidase.

### Activation of the N-ent loop mutant requires distinct internal cleavages of the pro-peptide at near neutral (pH 6.0) and acidic (pH 3.0) conditions

**Figure 3:**
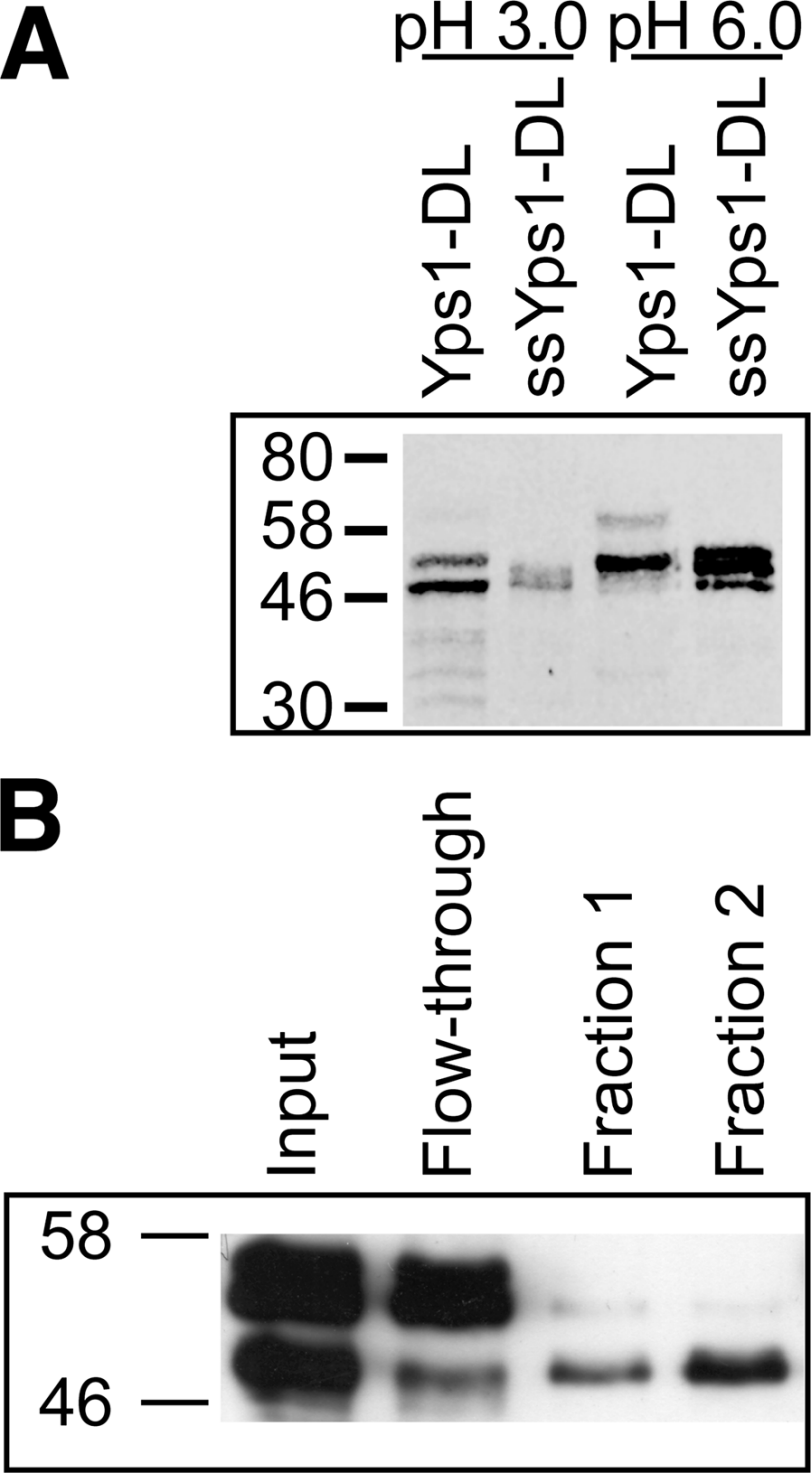
**Partial pro-peptide removal is essential for binding of Yps1-DL to a Pepstatin A-agarose resin. A)** Yeast *yps1Δ* cells transformed with the indicated plasmids were grown at either pH 3.0 or pH 6.0 for 24 h and equal amounts of concentrated media (7.7 × 10^7^ cells equivalent for Yps1-DL and 7.7 × 10^6^ cells equivalent for ssYps1-DL) were treated with Endo H_f_ and the proteins were separated by 10% SDS-PAGE. After transfer to PVDF membranes, immunoblotting was performed with an Yps1 antiserum (268–6) (Representative western from three independent experiments). **B)** Yeast *yps1Δ* cells transformed with ssYps1-DL were grown 24 h at pH 6.0 and the concentrated supernatant was applied to a Pepstatin A-agarose resin as described in “Methods”. Aliquots of the starting material (*Input*), the unbound material (*Flow-through*) and the eluted fractions (*Fraction 1 & 2*) were then separated by 10% SDS-PAGE and immunoblotted with an Yps1 antiserum (268–6) (Representative western from two independent experiments).

Deletion of the N-ent loop is expected to generate a single chain endopeptidase. To confirm this hypothesis and to analyze the zymogen activation of this mutant form of Yps1, a secreted soluble truncated version of Yps1-DL (ssYps1-DL) ending with a 6 × His-tag at Y^502^, as in ssYps1 (Figure 1A), was constructed. The Yps1-immunoreactive material secreted from *yps1Δ* yeast overexpressing ssYps1-DL was then compared to that resulting from the shedding of the *yps1Δ* yeast overexpressing Yps1-DL from 24 h cultures in unbuffered (referred to as pH 3.0, but the pH drops from 3.6 at start to slightly below 3.0 at the end of the incubation) and buffered (pH 6.0) SC -ura medium (Figure 3A). At pH 3.0, two main forms of 48- and 53 kDa were found in the supernatant of yeast expressing Yps1-DL, but some minor species suggestive of partial degradation were also detected below the 46 kDa marker. Two species were also recovered in the supernatant of yeast expressing ssYps1-DL (52 kDa and 48 kDa). The 48 kDa species from both ssYps1-DL and Yps1-DL were shown to possess an identical N-terminus (*see below*). Given that the 6 × His-tag is C-terminal to Y^502^ in ssYps1-DL (Figure 1A; numbering from full-length Yps1p), this therefore strongly suggests that shedding of the GPI protein Yps1-DL occurred through cleavage after K^498^ (numbering from full-length Yps1p) as previously determined for the native endopeptidase [8]. At pH 6.0, a minor form of ~66 kDa and a major 53 kDa form were detected in the medium of yeast overexpressing Yps1-DL. The secreted ~66 kDa species suggests that at pH 6.0 shedding also occurred further downstream from K^498^, but the precise C-terminal end is not known. In contrast, three molecular species of ~54-, ~52- and 48 kDa were immunodetected in the supernatant of yeast expressing ssYps1-DL. Purification by NiNTA affinity column of these three His-tag species followed by N-terminal sequencing (Table 1) indicated that the ~54 kDa form begins with K^22^ (*i.e.* has retained the entire pro-peptide), the ~52 kDa form begins with D^31^ (*i.e.* revealing internal cleavage of the pro-peptide after the basic doublet KR^30^) and the ~48 kDa species starts with A^68^ (*i.e.* complete pro-peptide removal). Because a unique sequence was obtained for each of these purified proteins, and at the very N-terminus, this proved that ssYps1-DL produces a single polypeptide chain endopeptidase.

**Table 1:** **N-terminal sequencing of purified Yps1 species**

**Sequenced sample**	**Culture medium pH**	**Molecular mass (kDa)**	Identified residues at each cycle*	**Deduced sequence**
*yps1∆* Yps1-DL	3.0	48	A^1^D^2^G^3^Y^4^E^5^E^6^I^7^I^8^I^9^T^10^N^11^Q^12^Q^13^S^14^F^15^	A^68^DGYEEIIITNQQSF
*yps1∆* ssYps1-DL	6.0	48	A^1^D^2^G^3^Y^4^E^5^E^6^I^7^I^8^I^9^T^10^N^11^Q^12^Q^13^S^14^F^15^	A^68^DGYEEIIITNQQSF
*yps1∆* ssYps1-DL	6.0	52	D^1^D^2^D^3^S^4^N^5^S^6^K^7^F^8^V^9^K^10^L^11^P^12^F^13^H^14^(K)^15^	D^31^DDSNSKFVKLPFHK
*yps1∆kex2∆* ssYps1-DL	6.0	54	K^1^I^2^I^3^P^4^A^5^A^6^N^7^K^8^R^9^D^10^D^11^D^12^S^13^N^14^S^15^	K^22^IIPAANKRDDDSNS

*:number in exponent matches the cycle number.

When applied to a Pepstatin A-agarose column (Figure 3B), the 48 kDa band nearly quantitatively absorbed to the affinity column, the 52 kDa form bound much less efficiently with the column whereas no binding was detected with the 54 kDa species from ssYps1-DL. Since Pepstatin A is an aspartic peptidase inhibitor acting as a *pseudo* substrate, these results indicate that access to the active site cleft of ssYps1-DL requires at least partial removal of the pro-peptide and that its complete removal significantly facilitates substrate/inhibitor binding. To identify the N-termini of the shed species from yeast expressing Yps1-DL, the 48- and 53 kDa forms observed at pH 3.0 were purified by Pepstatin A-agarose, separated by SDS-PAGE in the presence of DTT, transferred onto a PVDF membrane and each band was subjected to N-terminal sequencing. The N-terminal of the fastest migrating form (48 kDa) was shown to start with A^68^ (Table 1) but attempts to sequence the 53 kDa band were unsuccessful.

All together, we thus conclude that replacement of the Yps1 N-ent loop by the short peptide of Yps3 gives rise to a single chain endopeptidase, as intended, and that depending on the extracellular pH, complete (pH 3.0) or only partial (pH 6.0) pro-peptide removal is taking place. Although the exact N-terminus of the major shed species at pH 6.0 could not be determined, its binding to the Pepstatin A-agarose column (data not shown) and its migration (53 kDa) in between the 52 kDa and 54 kDa species characterized for ssYps1-DL strongly supports that an internal cleavage of the pro-peptide had occurred.

### Removal of the N-ent loop increases Yps1 shedding activity

**Figure 4:**
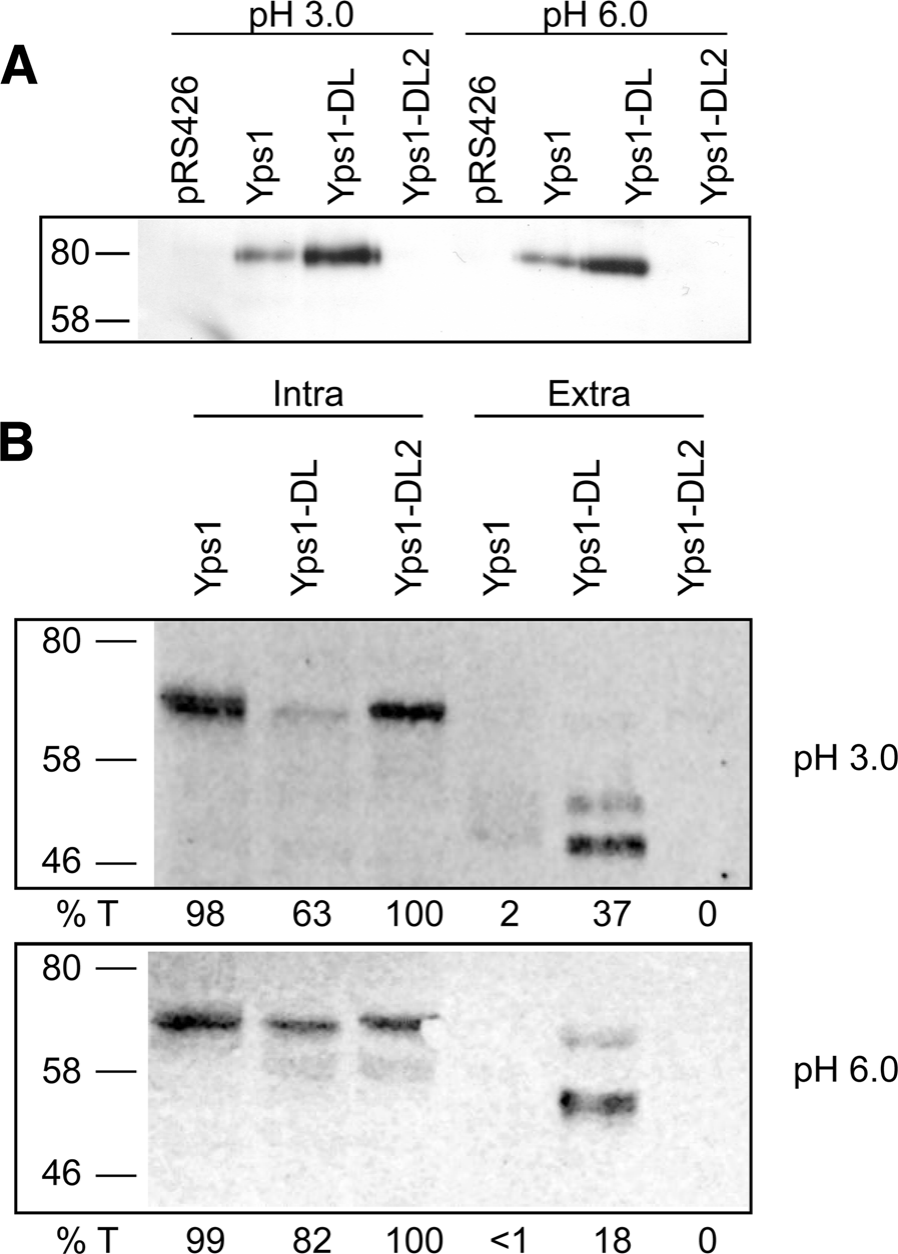
**Yps1-DL has increased shedding activity toward itself and Gas1 compared to Yps1. A)** Yeast *yps1Δ* cells transformed with the indicated plasmids were grown 24 h at either pH 3.0 or pH 6.0. Equal amounts of the concentrated supernatants, without prior treatment with Endo H_f_, were applied to 10% SDS-PAGE and immunoblotted with a Gas1 antiserum. **B)** Cell extracts (*C*) and concentrated supernatants (*S*), treated with Endo H_f_, were prepared from 24 h cultures at either pH 3.0 or pH 6.0 of *yps1Δ* yeast transformed with the indicated plasmids. After 10% SDS-PAGE and transfer to nitrocellulose membranes, proteins were immunodetected with an Yps1 antiserum (268–6). Note that 10-times more material was loaded for the extracellular material compared to cell extracts (Representative western from two independent experiments). The percentage of total Yps1-immunoreactive material (*%T*) found in cell extracts and supernatants is shown below the western blots.

The N-ent loop is proposed to shield the large S3 subsite of the binding pocket in fungal aspartic peptidases and to restrict access to the non-prime positions [13-17]. As known cleavage sites for Yps1 shedding of Gas1 (*T*G**K**^353^) and Yps1 itself (*S*AV**K**^498^) [8] are potentially *O*-glycosylated at the P3 or P4 position, respectively, it is conceivable that the N-ent loop partially mask or filter entry of these substrates into the catalytic site thus considerably restricting the efficiency of cleavage under normal conditions. To evaluate the role of this loop in Yps1 shedding activity, we compared the release of Gas1 and either Yps1 or Yps1-DL at both pH 3.0 and pH 6.0 (Figure 4A and B).

As previously reported, no Gas1 was found in the extracellular milieu from *yps1Δ* yeast expressing the vector alone (pRS426) at both pH 3.0 and pH 6.0. The overexpression of native Yps1 or Yps1-DL released an 80 kDa species of Gas1 into the medium whereas no Gas1 immunoreactive forms could be detected upon expression of the active site mutant Yps1-DL2 (Figure 4A). As estimated by image processing of the bands using ImageJ (http://rsbweb.nih.gov/ij/), approximately 2.5-fold (pH 3.0) and 2.2-fold (pH 6.0) more Gas1 was shed by Yps1-DL compared to native Yps1. Since both Yps1 and Yps1-DL are expressed from the same vector, suggesting that similar amounts are being produced, this provided a first indication that deletion of the N-ent loop increases the shedding activity of the endopeptidase.

To confirm that equivalent amounts of endopeptidase are produced by Yps1 and Yps1-DL and to compare their shedding activity toward themselves, the Yps1 immunoreactive material was analyzed both intra- and extracellularly using the Odyssey® Fc Imaging System to facilitate quantification (Figure 4B). At pH 3.0, a band of ~66 kDa was observed intracellularly and shed species of 53- and 48 kDa were detected in the supernatant from *yps1Δ* yeast expressing Yps1. Nearly 98% of the total Yps1 immunoreactive material was found intracellularly compared to 2% in the medium. For *yps1Δ* yeast expressing Yps1-DL, 37% of the Yps1 53- and 48 kDa shed species was found in the medium, an 18-fold increase compared to the shedding of Yps1. In yeast expressing the active site mutant of Yps1-DL (Yps1-DL2), all Yps1 immunoreactive material was found in the intracellular 66 kDa species (Figure 4B *top panel*) and this amounted to approximately 81% of the total (intra- and extra-cellular) estimated immunoreactive material for yeast expressing Yps1. Hence, Yps1 and Yps1-DL2 are expressed at similar levels. Therefore, the poor recovery of total immunoreactive material (intra- and extracellular) measured for Yps-DL (23% to that observed with Yps1) indicates that significant amounts were degraded upon release into the medium as already suggested from the presence of multiple faint species below the 46 kDa marker in Figure 3.

A similar pattern was observed at pH 6.0, except that the 48 kDa shed species was absent and, in addition to the 53 kDa species, Yps1-DL released a faint band of ~66 kDa (Figure 4B *bottom panel*) that suggests proteolytic processing downstream of K^498^, most likely C-terminally to KR^547^. Shedding of Yps1 was less efficient at pH 6.0 as evidenced by the recovery of 18% of the Yps1-immunoreactive material in the medium compared to 37% at pH 3.0 for yeast expressing Yps1-DL. The total Yps1-immunoreactive material associated with Yps1-DL and Yps1-DL2 at pH 6.0 (intracellular and extracellular) accounted for 59% and 51%, respectively, to that recovered for native Yps1. The lower recovery of intracellular Yps1-DL2 at pH 6.0 compared to that measured at pH 3.0 is obscure, but was observed in two independent experiments. Importantly, the shedding observed with Yps1-DL is largely if not exclusively Yps1-dependent as no species were released into the medium with the active site mutant Yps1-DL2 at either pH. Therefore, yeast expressing the N-ent loop mutant produced slightly less amounts of endopeptidase compared to native Yps1, particularly at pH 6.0, but the Yps1-dependent shedding was either strongly (Gas1) or drastically (Yps1) increased at both pH. This indicates that the N-ent loop plays a significant role in the Yps1 shedding activity.

**Figure 5:**
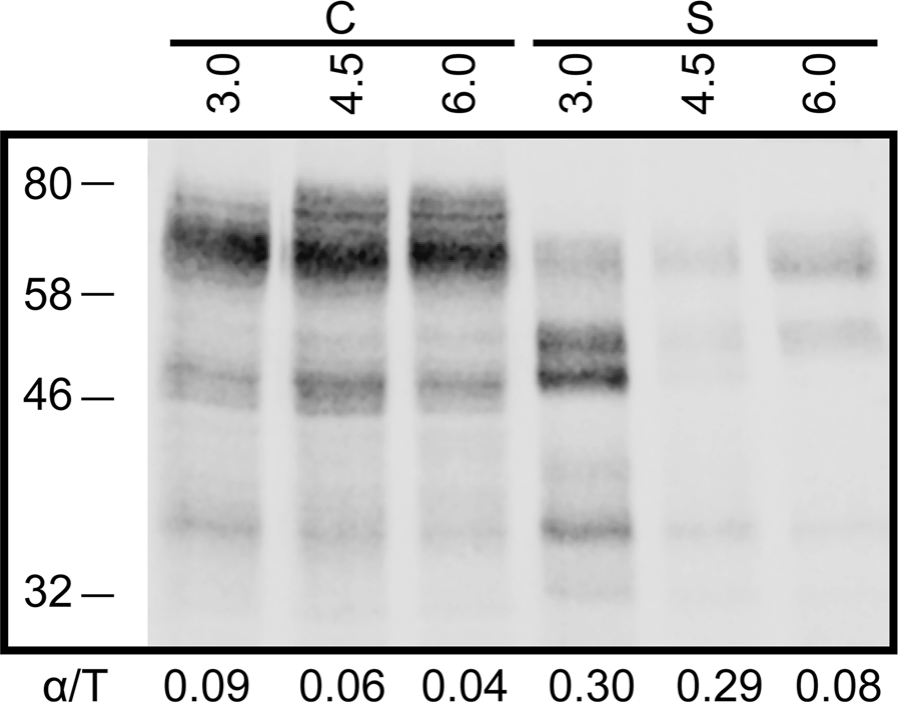
**Two-subunit Yps1 is enriched in the secreted shed species compared to the cell-associated forms.** Yeast *yps1Δ* cells transformed with plasmid Yps1 was grown 24 h at the indicated pH and aliquots from the cell extract (*C*; 5 × 10^6^ cells equivalent) and concentrated supernatant (*S*; 10-times more material compared to cell extract) were treated with Endo H_f_, separated by 10% SDS-PAGE and immunodetected with an Yps1 antiserum recognizing both the β and α subunits (294–3). The ratio of the immunoreactive Yps1 α subunit over the total Yps1 immunoreactive material (*α/T*) is shown at the bottom of each lane.

We hypothesized that cleavage of the N-ent loop in native Yps1, to generate the two-subunit endopeptidase, could stimulate its activity as observed for the N-ent loop mutant and that it might be intimately linked to its shedding from the cell surface. If this hypothesis is correct, it predicts that the ratio of immunoreactive material associated with the α subunit over the total Yps1 immunoreactive material (α/T) should be significantly greater in the medium (shed species) than inside the cells for native Yps1. This was tested under various pH conditions (pH 3.0, 4.5 and 6.0). As shown in Figure 5, the intracellular Yps1-immunoreactive material was resolved into a major broad species around 66 kDa (which contains a mixture of single-chain Yps1 and presumably some processed β subunit) and minor species of 53, 48 and 34 kDa; the latter corresponding to the α subunit [8]. The measured intracellular α/T ratios did not exceed 0.09 across the different pH conditions. In contrast, the extracellular α/T ratios were significantly greater than the cell associated ratios under acidic conditions (0.30 and 0.29 at pH 3.0 and 4.5, respectively), but not at pH 6.0 (0.08). This therefore supports the hypothesis that the shedding activity of native Yps1 is stimulated by internal cleavage of the N-ent loop, at least under acidic conditions (see Discussion).

### Hypoglycosylation of Gas1 and Yps1 in S/T rich domains or clusters stimulates their shedding from the cell surface

**Figure 6:**
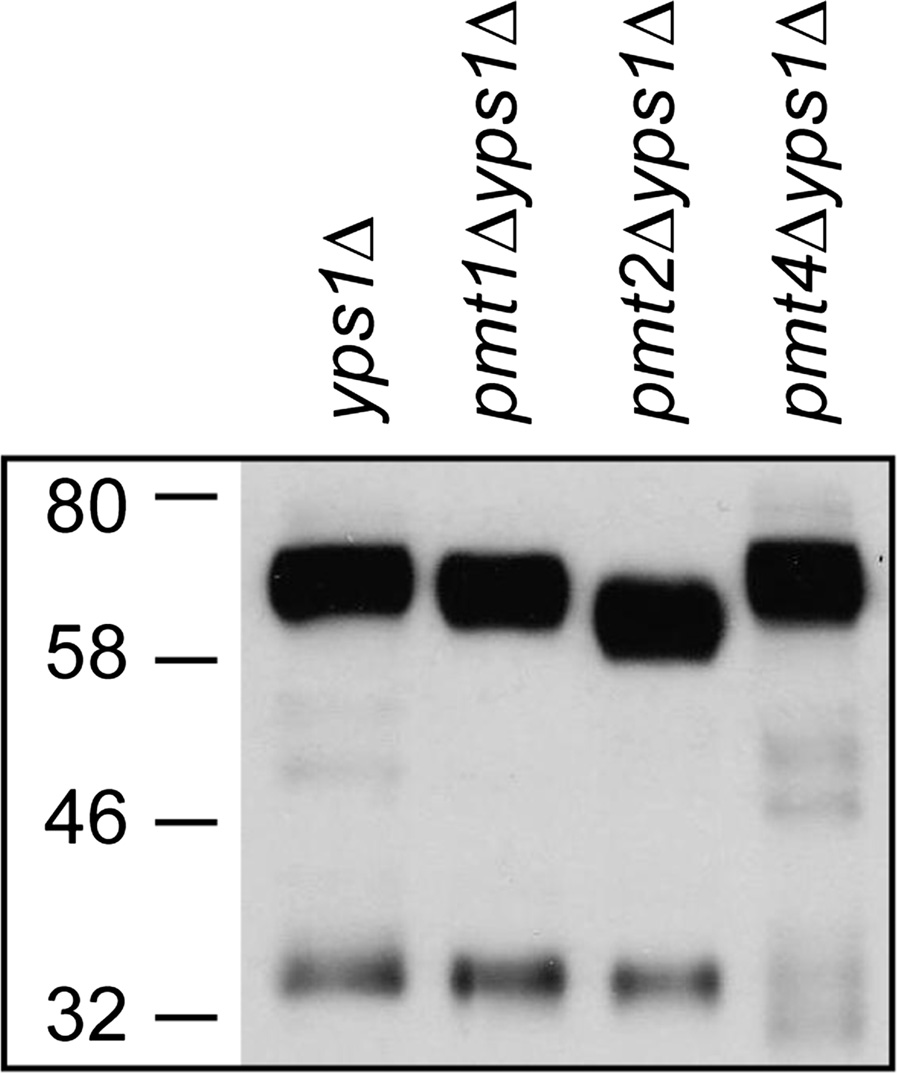
***O*** **-glycosylation of the Yps1 S/T-rich domain involves the Pmt2** ***O*** **-mannosyltransferase.** Yeast simple and double mutants transformed with Yps1 were grown 24 h at pH 3.0 and equal amounts of cell extracts treated with Endo H_f_ were analyzed by 10% SDS-PAGE and immunoblotted with an Yps1 antiserum recognizing both the β and α subunits (294–3).

As illustrated with Yps1 and Gas1 (see below), many potential or known cleavage sites for Yps1 are located in S/T rich domains or clusters that could be subjected to extensive *O*-glycosylation by one of the *S. cerevisiae* Pmt enzymes. Addition of mannose residues in close vicinity of the putative cleavage sites could hamper endoproteolysis through steric hindrance (for instance, when occupying the P4 and P3 positions with an intact N-ent loop in place). We thus explored what would be the consequence of preventing or reducing *O*-glycosylation of these model GPI proteins on their shedding by native Yps1 and the N-ent loop mutant. Toward this goal, we first investigated which Pmt enzyme was responsible for *O*-glycosylation of native Yps1 as assessed by gel mobility shift of Yps1 in cell extracts from *yps1Δ*, *yps1Δ pmt1Δ, yps1Δ pmt2Δ* or *yps1Δ pmt4Δ* mutants. As shown in Figure 6, the absence of Pmt2 had the most pronounced effect on the major 66 kDa band that shifted to ~58 kDa. The migration of the truncated version of Yps1 ending at position Y^502^ (ssYps1) was however not altered by the deletion of *PMT2* (not shown). In contrast, deletion of *PMT4* modified the migration of the 34 kDa α subunit. Therefore, the C-terminal S/T rich region of native Yps1 appears to be modified mostly, if not exclusively, by the Pmt2 *O*-mannosyltransferase.

**Figure 7:**
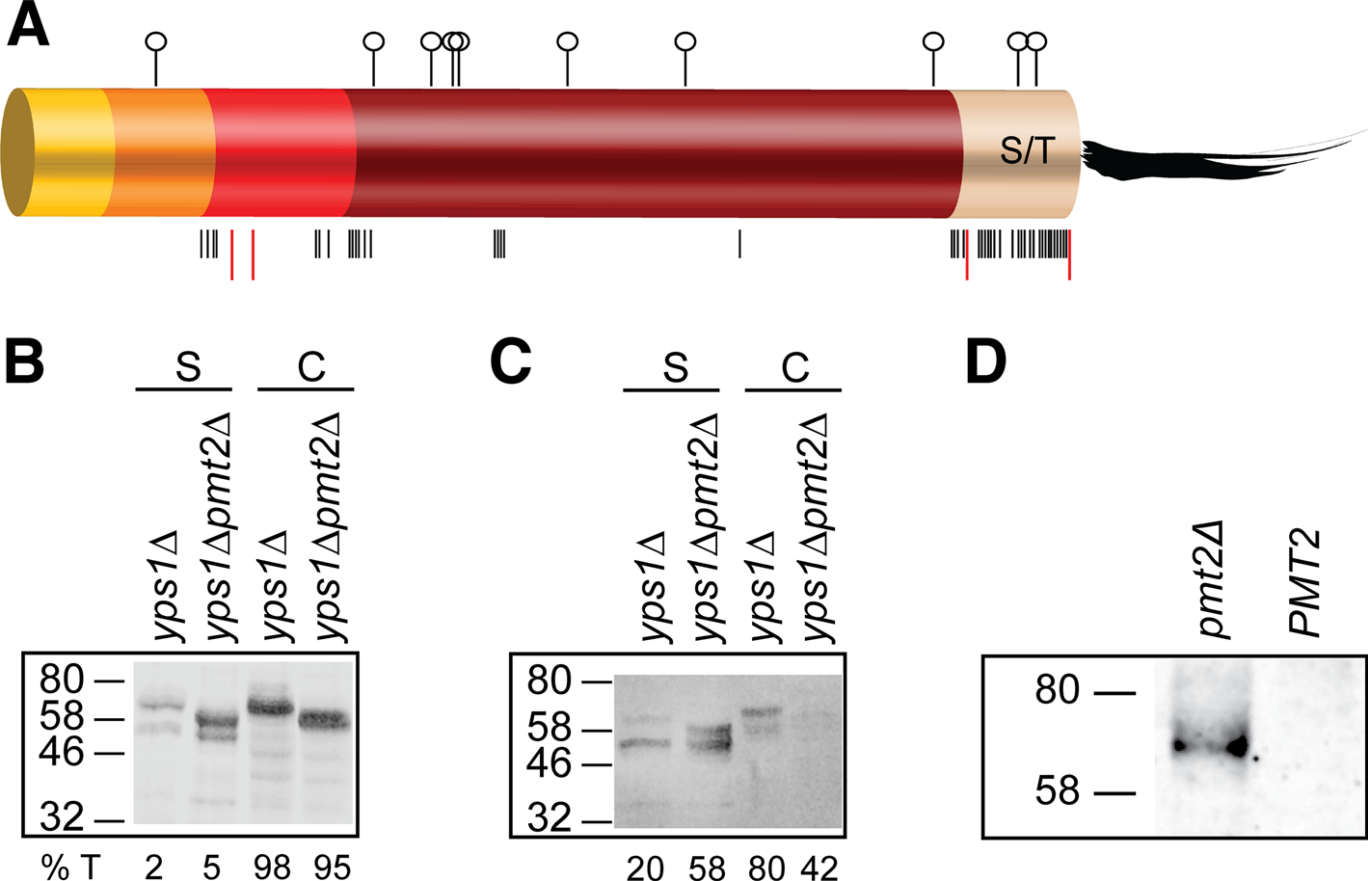
**Hypoglycosylation of Yps1 stimulates shedding of both native Yps1 and Yps1-DL. A)** Schematic view of Yps1 glycosylation profile and cleavage sites. Potential *O*-glycosylation sites, as determined by the NetOGlyc 4.0 algorithm (http://www.cbs.dtu.dk/services/NetOGlyc/) [23], are indicated with black bars whereas putative and/or known cleavage sites within these regions are illustrated by red bars. *N*-glycosylation sites are represented with a symbol. **B)** Cell extracts (*C*) and concentrated supernatants (*S*; 20-times more material compared to cell extracts) from 24 h cultures at pH 6.0 of the indicated yeast mutants transformed with plasmid Yps1 were treated with Endo H_f_, separated by 10% SDS-PAGE and immunodetected with an Yps1 antiserum (294–3). **C)** Cell extracts (*C* = 1.23 × 10^7^ cells equivalent) and concentrated supernatants (*S*; 5-times more material compared to cell extracts), from 24 h cultures at pH 6.0, of the indicated yeast mutants transformed with Yps1-DL were treated with Endo H_f_, fractionated by 10% SDS-PAGE and immunoblotted with an Yps1 antiserum (268–6). The percentage of total Yps1-immunoreactive material (*%T*) associated with cell extracts and supernatants is indicated for both Yps1 and Yps1-DL transformed yeast. **D)** Concentrated supernatants from 24 h cultures at pH 3.0 (5.6 × 10^7^ cells equivalent) of the indicated yeast mutants transformed with the catalytically inactive form of Yps-DL (Yps1-DL2) and analyzed as in **C)**.

Only two basic sites, K^498^ and KR^547^, are present within the Yps1 C-terminal S/T rich domain (Figure 7A) and whether hypoglycosylation of this region affected their use as cleavage sites by native Yps1 and Yps1-DL was tested in a *pmt2* background. As shown in Figure 7B, compared to that observed in *yps1Δ*, expression of native Yps1 in *yps1Δ pmt2Δ* yeast at pH 6.0 resulted in a 2.6-fold increase in the amount of the 53 kDa shed form (cleavage after K^498^) in the medium. It also led to both an increase (2.9-fold) and a shift, resulting from the absence of *O*-glycosylation, of the ~66 kDa species to 58 kDa (cleavage after KR^547^). For Yps1-DL, the absence of Pmt2 had a similar effect on the relative abundance of the 53 kDa and 58 kDa species found in the medium (Figure 7C; 2.9-fold increase with 58% of the total Yps1 immnoreactive material recovered in the medium). Hence, although shedding of Yps1-DL at pH 6.0 was already significantly more efficient than that observed for native Yps1 (mean values of 19% vs 1% from Figures 4 and 7), deletion of *PMT2* enhanced shedding of both native Yps1 and Yps1-DL. However, hypoglycosylation of Yps1, in addition to stimulate its self-shedding, also led to some release of the catalytically inactive Yps1-DL2 mutant into the medium (Figure 7D). Therefore, hypoglycosylation of the S/T rich region of Yps1 makes this domain more susceptible to proteolytic processing by both Yps1 and other peptidases, possibly other yapsin members. Similar results were obtained at pH 3.0, except that as much as 23% of native Yps1 was shed from the cell surface in the *pmt2* background and Yps1-DL, under these conditions, was nearly entirely shed from the cell surface (not shown).

**Figure 8:**
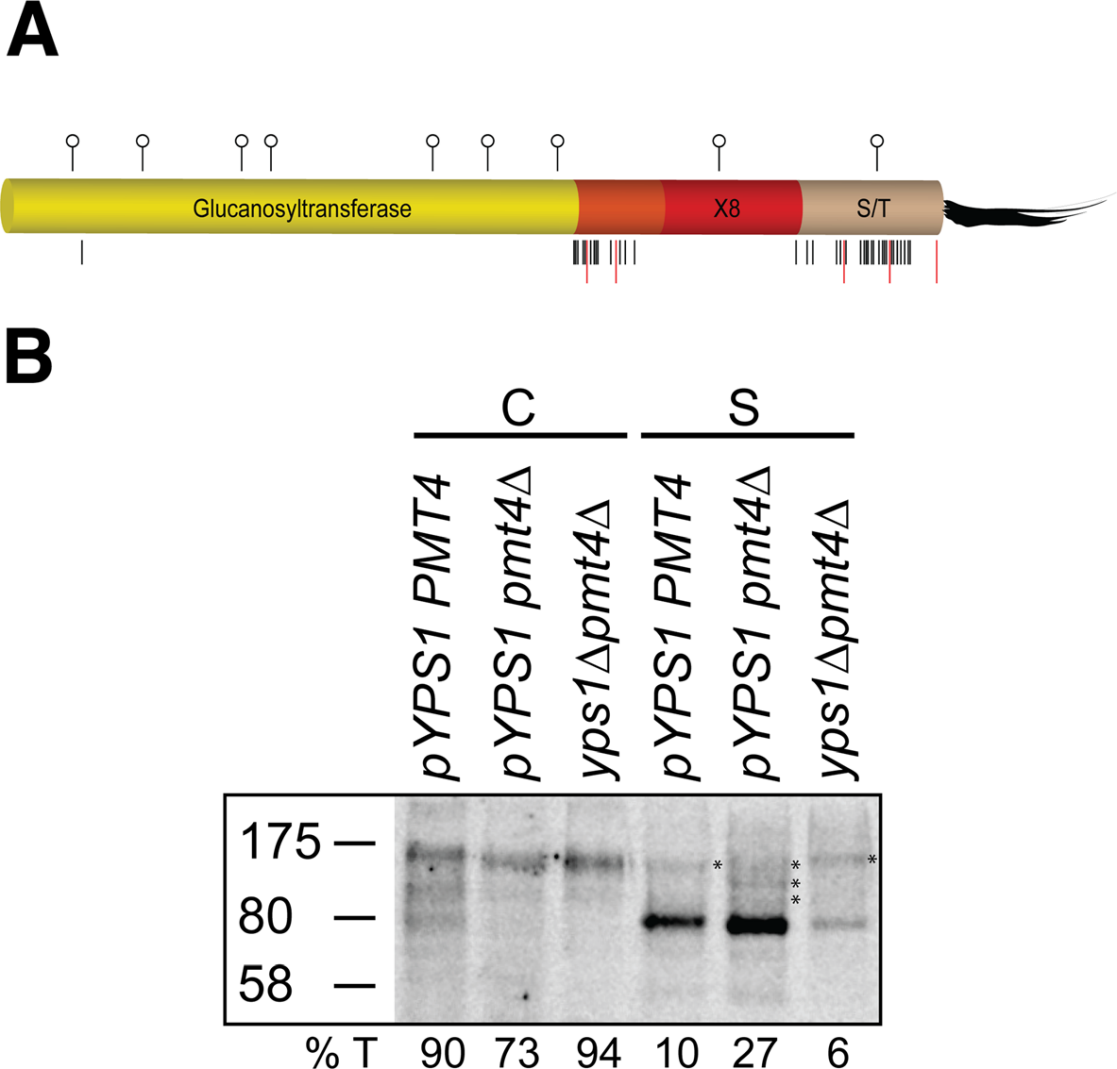
**Hypoglycosylation of Gas1 stimulates shedding by native Yps1. A)** Schematic view of Gas1p glycosylation profile and putative cleavage sites as depicted for Yps1 (Figure 7A). **B)** Cell extracts (*C*; 5.0 × 10^6^ cells equivalent) and concentrated supernatants (*S*; 10-times more material compared to cell extracts) from 24 h cultures at pH 6.0 of the indicated yeast mutants (all strains are also *yps1Δ*) either transformed (pYPS1) or untransformed with plasmid Yps1 were separated by 10% SDS-PAGE and immunodetected with a Gas1 antiserum. Asterisks point to faint discrete Gas1 species detected above the major 80 kDa species in the extracellular milieu. The percentage of total Gas1-immunoreactive material (*%T*) associated with cell extracts and supernatants is indicated for all strains.

As shown in Figure 8A, the potential *O*-glycosylation sites predicted for Gas1 are grouped in two regions, the inter-domain separating the Glucanosyltransferase domain from the X8 domain and the C-terminal S/T rich domain. In addition to the previously determined cleavage site for Yps1 (K^353^) located within the inter-domain [8], several basic sites are present in these two regions. Wild type Gas1 is known to be *O*-mannosylated by Pmt4 [24] and its deletion led to the release of 27% of Gas1 into the medium compared to 10% in *PMT4* expressing native Yps1 (Figure 8B). While the 80 kDa species was the predominant form accumulating in the medium, several larger species could also be detected (*asterisks* in Figure 8B) in the *pmt4* background. As observed with Yps1-DL (Figure 7D), hypoglycosylation of Gas1 also resulted in some Yps1-independent shedding (6%). We thus conclude from these experiments that *O*-mannosylation is a determinant factor regulating Yps1-dependent and -independent shedding of model GPI proteins.

## Discussion

Most aspartic endopeptidases are initially synthesized as inactive zymogen requiring proteolytic removal of a pro-peptide to become active [10]. Using the single-subunit Yps1 endopeptidase Yps1-DL, we showed here that pro-peptide removal is pH dependent. At pH 3.0, the GPI-anchored form of Yps1-DL was proteolytically processed to generate a mature endopeptidase starting with A^68^, implying cleavage C-terminal to the KR^67^ basic residues as previously reported for a truncated secreted soluble version of Yps1p [8,11]. Although attempts to determine the exact N-termini of Yps1-DL at pH 6.0 were unsuccessful, we demonstrated that the endopeptidase was active at this pH and that only forms missing parts of the pro-peptide could bind to Pepstatin A-agarose column. Given the presence of numerous basic residues within the Yps1 pro-peptide, it is most likely that an intramolecular processing event occurs C-terminal to one of these basic residues at pH 6.0, possibly K^37^ as originally proposed for the truncated version of the enzyme [11].

Yps1 substrate specificity is relatively broad, pair basic residues and single basic residues [3-6]. Basic residues are numerous within the S/T rich regions of Utr2p, Gas1p and Yps1p, yet Yps1p appears to cleave significantly these substrates only under stressful conditions [9]^a^. We hypothesized that the N-ent loop acts as a filter to restrict hydrolysis of putative cleavage sites present in numerous cell surface proteins. Replacement of the large Yps1p N-ent loop between Cys^117^ and Cys^186^ by the short peptide found at the same location in Yps3p was shown here to stimulate its shedding activity either strongly (Gas1) or dramatically (Yps1). The difference in shedding of Gas1 vs Yps1-DL most likely reflects the fact that the endopeptidase (Yps1-DL) is removed so efficiently from the cell surface that contact between GPI-anchored Gas1 and Yps1-DL is only transient. Importantly, replacement of the loop did not modify the specificity of the enzyme as the same cleavage sites appeared to be used for Yps1 and Yps1-DL. We earlier proposed that cleavage within the N-ent loop might facilitate the shedding activity of the enzyme [8]. As shown here with the native Yps1 enzyme, 4 to 5-fold more endopeptidase was detected into two-subunits in the medium than inside the cells under acidic conditions (pH 3.0 and pH 4.5), which gives credence to this hypothesis. The finding that this correlation was not as strong at pH 6.0 is puzzling. However, significantly more shedding of a catalytic inactive mutant of native Yps1 (D^101^A) was observed at pH 6.0 than at pH 3.0 [8]. This suggests that the lower ratio of two-subunit Yps1 observed in the medium at pH 6.0 is due, at least partly, to Yps1-independent shedding of single-chain Yps1. Hence, the N-ent loop indeed seems to restrict access to the active site of the endopeptidase and stabilizes the enzyme at the cell surface. Its deletion or internal cleavage is associated with an increase shedding activity highlighting its regulatory role in Yps1 function. It is thus plausible that cleavage of the Yps1 N-ent loop is stimulated under stressful conditions and further studies should address this important issue.

Shedding of Yps1 substrates typically takes place in S/T rich regions that are predicted to be heavily decorated by *O*-glycosylation. In Yps1, the *O*-mannosyltransferase Pmt2 is preferentially responsible for the addition of mannose residues within the C-terminal S/T rich domain. For wild type Gas1, Pmt4 is involved but neither Pmt1 nor Pmt2 [24]. We showed here that the absence of Pmt2 stimulates the shedding of both native Yps1 and the N-ent loop mutant Yps1-DL whereas the absence of Pmt4 increases shedding of Gas1. While this increase shedding was largely Yps1-dependent, in both cases we provided evidence for the involvement of other endopeptidases as well, possibly other yapsin members. It therefore suggests that the extent of *O*-glycosylation is a major determinant for the stability of cell surface proteins by protecting against Yps1-dependent and -independent shedding activities. In line with this hypothesis, it is worth noting that the absence of *O*-glycosylation leads to aberrant proteolytic processing of the cell surface sensors Mid2 and the Wsc proteins [18]. Furthermore, numerous heterologously expressed proteins are subjected to Yps1 proteolysis [1]. For the heterologous proteins expressed in yeast, the cleavage sites used are not within typical S/T rich regions found in yeast surface proteins and may therefore be hypoglycosylated. Of much interest, differential *O*-glycosylation at specific sites is now emerging as an important regulator of processing by the mammalian pro-protein convertases [25]. In light of the present study, the function of yeast yapsins may similarly be regulated by differential *O*-mannosylation resulting, for instance, from stressful conditions.

That hypoglycosylation affects the stability of yeast cell surface proteins through shedding suggests that it may be linked with the elimination of misfolded proteins that escaped the ER quality control. Pmt2 was recently reported to play an important function in the ER-associated degradation (ERAD) pathway of unfolded proteins [26,27]. Specifically, it was demonstrated that futile cycles of unsuccessful folding ends up with the Pmt2-catalyzed *O*-glycosylation that in turns directs the unfolded protein to the ERAD pathway. Hence, the absence of Pmt2, or an excess of Pmt2 substrates, likely results in the accumulation of misfolded or unfolded proteins, some escaping ERAD to reach the cell surface. Indeed, the absence of Pmt2 was shown to promote post-ER degradation of a model misfolded protein, Gas1*, which in contrast to native Gas1 is a substrate for Pmt2 [26,28]. Deletion of the vacuolar peptidase Pep4 was found to delay, but not eliminate, degradation of Gas1* suggesting that the vacuole may only be one of the alternative mechanisms [28]. In light of the data observed with Yps1 in the *pmt2* mutant, it is plausible that shedding at the cell surface by Yps1-dependent and Yps1-independent processing may as well contribute. We are currently addressing more directly this hypothesis through proteomic analysis of the secretomes from yeast carrying or not functional *PMT* and/or *YPS* genes.

## Conclusions

Replacement of the N-ent loop of Yps1 by the short peptide found in the same position in Yps3, by producing a single chain endopeptidase, allowed us to elucidate the mechanism by which zymogen activation proceeds at distinct pH for this GPI-anchored endopeptidase. Partial pro-peptide removal is a necessary and critical step to produce a functional enzyme. Without its N-ent loop, the Yps1 shedding activity is strongly increased suggesting that this domain acts as a major determinant to restrict access to active site and that internal cleavage regulates its activity. *O*-glycosylation was also shown to protect against endoproteolysis by native or single-chain Yps1, and possibly other yapsin members. We postulate that misfolded proteins that escape ERAD might be hypoglycosylated, which would trigger their elimination at the cell surface by Yps1 and/or other yapsin members.

## Methods

### Bacterial and yeast strains and growth media

The strain of *E. coli* used in this study was MC1061. Bacteria were grown in 2 × YT medium (1% Yeast extract, 1.6% Tryptone and 0.5% NaCl) with 0.2% dextrose and 100 μg/ml ampicillin to maintain plasmid selection [29]. All *S. cerevisiae* strains used in this study (Table 2) were isogenic to BY4741 (MAT**a***ura3 his3 leu2 met15)* (Open Biosystems). To construct *yps1ΔpmtΔ* double mutants, Matα *yps1Δ* was crossed with the appropriate Mat**a***pmtΔ* yeast, the resulting diploids sporulated, and ascospores from non-parental ditype tetrads resistant to 0.25 μg/ml G418 sulfate (Calbiochem) were selected. The presence of the *yps1* and *pmt* mutated alleles of each haploid double mutant was confirmed by PCR with the corresponding PMTX F and KANB oligonucleotide primers, shown in Table 3. Yeast strains were grown in YPD (1% Yeast extract, 2% Peptone and 2% Dextrose) or SC (synthetic complete) (0.67% Yeast nitrogen base without amino acid and 2% Dextrose) lacking uracil to maintain plasmid selection [28]. Yeast cultures in SC-Ura were either buffered at pH 6.0 or pH 4.5 or unbuffered (pH 3.0). SDS sensitivity was tested by adding 0.01% SDS to YPD plates and plates were photographed after 2–3 days.

**Table 2:** **Yeast strains used in this study**

**Strain**	**Genotype**	**Source**
*yps1Δa*	*Mat* ***a*** *yps1Δ::KANMX6 ura3 his3 leu2 met15*	Open Biosystems
*yps1Δα*	*Mat* ***α*** *yps1Δ::KANMX6 ura3 his3 leu2 lys2*	Open Biosystems
*pmt1Δ*	*Mat* ***a*** *pmt1Δ ::KANMX6 ura3 his3 leu2 met 15*	Open Biosystems
*pmt2Δ*	*Mat* ***a*** *pmt2Δ::KANMX6 ura3 his3 leu2 met 15*	Open Biosystems
*pmt4Δ*	*Mat* ***a*** *pmt4Δ::KANMX6 ura3 his3 leu2 met 15*	Open Biosystems
*yps1Δkex2Δ*	*Mat* ***a*** *yps1Δ::KANMX6 kex2Δ::KANMX6 ura3 his3 leu2*	[8]
*yps1Δpmt1Δ*	*Mat* ***a*** *yps1Δ::KANMX6 pmt1Δ::KANMX6ura3 his3 leu2 met15*	This study
*yps1Δpmt2Δ*	*Mat* ***a*** *yps1Δ::KANMX6 pmt2Δ::KANMX6ura3 his3 leu2 met15*	This study
*yps1Δpmt4Δ*	*Mat* ***a*** *yps1Δ::KANMX6 pmt4Δ::KANMX6ura3 his3 leu2 met15*	This study

**Table 3:** **Oligonucleotides used in this study**

**Name**	**Description**	**Sequence**
YPS1 F	Strain deletion confirmation	5′-CTTTTATACTGCGCCATGAGTAGTT-3′
PMT1 F	Strain deletion confirmation	5′-CTTCAATTACGCTTTCTACCAACAT-3′
PMT2 F	Strain deletion confirmation	5′-ACATATTATTGGACACGTCGCCC-3′
PMT4 F	Strain deletion confirmation	5′-TTCCTACTTGTCCTTTCTTTCCTTT-3′
KANB	Strain deletion confirmation	5′-CTGCAGCGAGGAGCCGTAAT-3′
Loop F	N-ent loop replacement in pRS*YPS1*	5′-[PO_4_]-GCTCGGATAATCCATACTGTGGT TCTGTGATGGACTGTCAACAATACGG-3′
YB E^101^-F	Mutation D^101^E	5′-GGTCCTGGTGGAAACAGGCTCCTCT-3′
YB E^101^-R	Mutation D^101^E	5′-AGAGGAGCCTGTTTCCACCAGGACC-3′
YB40	Amplification of pYPS1-DL for gap repair in pssYPS1	5′-TGGAAGTGGGCACGCCAC-3′
YB43	Amplification of pYPS1-DL for gap repair in pssYPS1	5′-GAACACTATTTCCATACT-3′

### DNA manipulations, plasmids and oligonucleotides

DNA manipulations, bacterial and yeast transformations were all carried out according to standard procedures [29,30]. Unless otherwise indicated, all restriction and DNA-modifying enzymes were purchased from New England Biolabs Ltd (Pickering, ON, Canada). Oligonucleotides were purchased from Eurofins MWG Operon and are listed in Table 3. Plasmids used in this study are listed in Table 4. pRSYPS1-DL (pYPS1-DL) is derived from pRSYPS1 (pYPS1) [8] in which the sequence encoding for Yps1p N-ent loop (Cys_117_-Cys_186_ Yps1p annotation) was replaced by the sequence encoding for the N-ent loop of Yps3p (Cys_97_-Cys_103_ Yps3p annotation). This construction was done using the Kunkel mutagenesis method [31,32] using the oligonucleotide Loop F. pssYPS1-DL is derived from pBA22-His (pssYPS1) [33] and was done by gap repair using a PCR fragment of pYPS1-DL with the oligonucleotides YB40 and YB43. QuickChange site-directed mutagenesis kit (Stratagene, La Jolla, CA) was used for the mutagenesis of plasmids pYPS1-DL and pssYPS1-DL to construct the corresponding active site mutants pYPS1-DL2 and pssYPS1-DL2 using the oligonucleotides YB E^101^-F and YB E^101^-R. All plasmid constructs were analyzed by DNA sequencing.

**Table 4:** **Plasmids used in this study**

**Plasmid**	**Description**	**Source**
pRS426	2 μ replication origin, *URA3* marker	[34]
pYPS1	pRS426 expressing the *YPS1* gene from its own promoter	[8]
pYPS1-C2	pYPS1 with D^101^E mutation	[8]
pYPS1-DL	pYPS1 in which the Yps1p N-ent loop is replaced by the Yps3p codons 98 to 102	This study
pYPS1-DL2	pYPS1-DL with D^101^E mutation	This study
pBA22H (pssYPS1)	2 μ replication origin, expressing a truncated version of Yps1p (Δ67 C-ter) with a 6×His-tag	[33]
pssYPS1-DL	pssYPS1 in which the Yps1 N-ent loop is replaced by the Yps3p codons 98 to 102	This study
pssYPS1-DL2	pssYPS1-DL with D^101^E mutation	This study

### Cellular extraction, culture medium concentration and immunodetection

Cellular extraction and culture medium concentration were done according to the methods described in [8]. Unless otherwise indicated, 3.6 × 10^6^ cells equivalent were used for total protein extraction. To reduce heterogeneity, all samples were treated with Endo H_f_ (New England Bioloabs) as described in [8] for Yps1 immunodetection. The treated samples were analyzed on 10% SDS-PAGE and transferred to either PVDF (GE Healthcare) or nitrocellulose membranes (Mandel Scientific). Samples on PVDF membranes were revealed with the ECL plus detection system (GE Healthcare) according to the manufacturer procedure. Proteins on nitrocellulose membranes were immunodetected using the Odyssey® Fc Imaging System (Li-Cor, Mandel Scientific) according to the manufacturer procedure. Nitrocellulose membranes were blocked with Odyssey® Blocking Buffer (Mandel Scientific) prior to incubation with the primary antibodies. Yps1-DL was detected using a rabbit Yps1 antiserum (Anti-268-6; 1/5000 dil) raised against amino acids 245 to 532 of Yps1^a^ whereas Yps1 was alternatively detected, as indicated, using Anti-294-3 (1/10000 dil) raised against amino acids 35 to 246 of Yps1 [35]. Gas1 was detected with a rabbit antiserum (1/5000 dil) generously provided by H. Riezman (University of Geneva). Secondary antibodies were either a horseradish peroxidase-conjugated anti-rabbit antibody (Sigma) for ECL detection or an 800 infrared dye-labeled anti-rabbit antibody (Mandel Scientific) for the Odyssey® Fc Imaging System used at the same final dilution as the primary antibodies.

### Protein purification

Purification of the truncated form of Yps1 (ssYps1-DL) with a NiNTA resin was done accordingly to the method described in [8]. Purification of Yps1-DL was done using a Pepstatin A-agarose resin (Sigma) with the procedure described by the manufacturer. Cleared supernatants from 24 h yeast culture (50 ml) were concentrated to 500 μl with Amicon Ultra 30 kDa (Millipore). The concentrated supernatants were diluted two times by adding 15 ml of binding buffer (100 mM Sodium citrate, 500 mM NaCl pH 3.0) and concentrated to 500 μl. Protein concentrates were applied to 100 μl of Pepstatin A-agarose for 3 h at room temperature. After 3 washes with 1 ml of binding buffer, proteins were eluted from the resin in two fractions of 500 μl with elution buffer (100 mM Sodium carbonate, 500 mM NaCl pH 8.7). Purifications were then analyzed on SDS-PAGE using 20 μl of each fraction.

### Edman degradation

Purified forms of ssYps1-DL or Yps1-DL were fractionated on SDS-PAGE and then transferred to PVDF membranes in 10 mM *N*-cyclohexyl-3-aminopropanesulfonic acid (CAPS) buffer pH 11 (Sigma). The membranes were then stained with 0.1% PhastGel Blue R (GE Healthcare), 40% methanol and 10% acetic acid and destained in 50% methanol. Bands of interested were cut out and subjected to automated Edman degradation for 15 cycles (Institut de recherches cliniques de Montréal, Montréal, Canada).

## Endnotes

^a^Dubé, AK, Coulombe, C, Bélanger, M, Gagnon-Arsenault, I, Fujita, M and Bourbonnais, Y (manuscript in preparation).

## Abbreviations

GPI: Glycosylphosphatidylinositol
N-ent loop: N-entrance loop
UPR: Unfolded protein response
SAP: Secreted aspartic peptidases
ERAD: ER-associated degradation
CAPS: *N*-cyclohexyl-3-aminopropanesulfonic acid
